# Functioning Nanomachines Seen in Real-Time in Living Bacteria Using Single-Molecule and Super-Resolution Fluorescence Imaging

**DOI:** 10.3390/ijms12042518

**Published:** 2011-04-15

**Authors:** Sheng-Wen Chiu, Mark C. Leake

**Affiliations:** 1 Biochemistry Department, South Parks Road, Oxford University, Oxford OX1 3QU, UK; E-Mail: sheng-wen.chiu@linacre.ox.ac.uk; 2 Clarendon Laboratory, Physics Department, Parks Road, Oxford University, Oxford OX1 3PU, UK

**Keywords:** fluorescence microscopy, fluorescent protein, *in vivo* imaging, molecular machine, nanomachine, photobleach, single molecule, slimfield, super-resolution, total internal reflection

## Abstract

Molecular machines are examples of “pre-established” nanotechnology, driving the basic biochemistry of living cells. They encompass an enormous range of function, including fuel generation for chemical processes, transport of molecular components within the cell, cellular mobility, signal transduction and the replication of the genetic code, amongst many others. Much of our understanding of such nanometer length scale machines has come from *in vitro* studies performed in isolated, artificial conditions. Researchers are now tackling the challenges of studying nanomachines in their native environments. In this review, we outline recent *in vivo* investigations on nanomachines in model bacterial systems using state-of-the-art genetics technology combined with cutting-edge single-molecule and super-resolution fluorescence microscopy. We conclude that single-molecule and super-resolution fluorescence imaging provide powerful tools for the biochemical, structural and functional characterization of biological nanomachines. The integrative spatial, temporal, and single-molecule data obtained simultaneously from fluorescence imaging open an avenue for systems-level single-molecule cellular biophysics and *in vivo* biochemistry.

## 1. Introduction

Biological machines at the nanometer length scale are molecular scale complexes, generally made up of multiple protein sub-units, which act as energy transduction devices that respond to specific biological stimuli to perform coordinated mechanical work [1–3]. The realization that a large collection of such dedicated polypeptide nanomachines carry out the various tasks in a living cell deeply alter our conventional view of living systems [1,4]. Optimized over an evolutionary time scale of up to 4 billion years, these sophisticated nanomachines are also the starting point for bionanotehcnology in constructing more powerful synthetic machines [2,3,5]. At the most fundamental level our understanding of these biological molecular machines is essential in exploring the inner workings of living cells [1].

Bacteria contain a huge number of nanomachines that display diverse functionalities. They can perform tasks involved in cell motility, chromosome and plasmid segregation, cytokinesis, DNA replication, energy generation, and protein synthesis and secretion, to name but a few [5–15]. As well-characterized experimental model organisms, bacteria are relatively easy to grow and manipulate, and are feasible for performing single-molecule and super-resolution fluorescence imaging [16]. Indeed, research focused on bacterial molecular machines has unveiled many mechanistic insights of how biological nanomachines in general work.

Even the simplest biological nanomachine is composed, in general, of a highly coordinated multiprotein assembly. Their dynamic stoichiometries and architectures, as well as forces and nanometer-scale conformational changes generated as part of their function, are all essential features that need to be investigated. Several different experimental methods have been applied in parallel in this regard. Traditional biochemical and biophysical analyses, electron microscopy, X-ray crystallography, nuclear magnetic resonance spectrometry and mass spectrometry, have all provided significant biochemical, biophysical, and structural data for biological nanomachines [17–19]. However, these techniques primarily deal with isolated protein complexes, many of which are only parts of the intact nanomachine. Consequently, information on the organization and interactions of the protein components, and their precise functions within the context of a fully functional nanomachine is lost [18]. Moreover, traditional biochemical and biophysical methods study the average behavior of a population, which might mask the full distribution of behavior and produce a misleading picture of a system; the mean average behavior is not necessarily equal to the total collective or integrated behavior over the whole system.

Single-molecule approaches provide fresh ways of observing the hidden world masked by ensemble averaging. The drawbacks of bulk ensemble-average approaches, and the advantages of single-molecule approaches are: (1) heterogeneity and stochasticity, two intrinsic features of biological systems, can only be revealed by studying single molecule events; (2) temporal averaging may blur novel features of a dynamic molecular process, including transient/rare events and their ordering in time and space; (3) perturbing synchronization is unnecessary for single-molecule studies as is often the case for ensemble level studies, for example to ensure that all cells in a population are in the same phase in their respective cell cycles; (4) the results of ensemble measurements can often be interpreted in multiple, indirect ways, whereas single-molecule studies in general provide a more definitive direct indication; (5) single-molecule approaches facilitate the direct quantitative measurement of critical properties of single biomolecules and their assemblies, including the forces, motions/steps, and conformational changes; (6) single-molecule approaches facilitate the ultimate miniaturization and multiplexing of biological assays [20–25].

Although there have been multiple ingenious technical approaches for performing single-molecule experiments, four such stand out in their recent popularity, to visualize, detect, and manipulate individual molecules: atomic force microscopy, laser tweezers, magnetic devices, and single-molecule fluorescence microscopy/spectroscopy [20,21,23,24,26]. The application of single-molecule techniques *in vitro* has greatly improved our knowledge of biological nanomachines [12,23,25,26]. However, *in vitro* studies focus on isolated molecules in artificial environments. The behavior of a molecular process may be different between *in vivo* and *in vitro* conditions [25,27–29]. The dynamics of nanomachines in their native environment, and the cooperation of them with the cellular functional networks cannot be readily obtained by purely *in vitro* approaches. Rather, if at all technically feasible, we need to measure the composition, organization and dynamics of molecular machines in functioning, living cells. Central to this challenge is single-molecule and super-resolution fluorescence imaging, which potentially offer nanometer-level spatial precision, *ca.* millisecond temporal resolution, single-molecule sensitivity, molecular specificity, multiplexing, and parallel data acquisition [27,30,31]. The fact that many biomolecules exist in low copy numbers in a living cell also necessitates single-molecule methods [22,31].

In this review, we highlight some of the methodological innovations and new observations from studies focused on functioning nanomachines in living bacteria using single-molecule and super-resolution fluorescence imaging. These examples illustrate the power of fluorescence imaging in unveiling the behaviors of functioning molecular machines in their true physiological context.

## 2. Single-Molecule Fluorescence Microscopy

### 2.1. Fluorescence Imaging: A Brief Introduction

Owing to its relative ease of implementation and minimally-perturbative/non-invasive nature, fluorescence microscopy [32] is among the most versatile and accessible method for direct observation. The richness of fluorophores and advances in labeling methods enable the simultaneous observations of the distributions and abundance of multiple specific molecules in living cells. The power of visual representation, characterization, and quantification makes fluorescence microscopy a central tool in biology.

The spatial precision of fluorescence microscopy can be described in terms lateral (*x*-*y*) and axial (*z*) spatial resolution, as well as localization accuracy. In simple terms, the spatial resolution is the minimum distance by which two objects can be distinguished, while localization accuracy is the minimum distance, or equivalent volume, with which one can locate an object’s position [33–36]. The temporal resolution is how fast images from a given system can be faithfully sampled. The spatial and temporal resolutions are determined by the whole imaging system, including the microscope, the light source, the detector, and the software [24,35,37,38]. To obtain informative live-cell fluorescence images, the choice of fluorophores and the preparation and incubation of cells are also important [24,35,37,38].

The main challenge of live-cell single-molecule fluorescence imaging is to enhance the signal-to-noise ratio [24,31,39]. This can be achieved by decreasing the cellular autofluorescence background or increasing detectable fluorescent signals. Autofluorescence is usually homogeneous in bacterial cells and can be significantly reduced by growing cells in a defined minimal medium [39]. Whenever possible, choosing fluorophores with red-shifted spectra is also helpful. Other methods to reduce background are pre-photobleaching and minimizing/delimiting the excitation volume [24,39], which are discussed below. It should be borne in mind that all images are subject to interpretation, and this is especially true for single-molecule and super-resolution fluorescence imaging which both heavily rely on image processing [16,24,34,35,37].

### 2.2. Fluorescent Proteins and Their Applications

The common fluorophores used in fluorescence microscopy are small organic fluorescent dyes, nanocrystals (quantum dots), autofluorescent proteins, and small genetic encoded tags complexed with fluorochromes [40,41]. Among them, autofluorescent protein (FP)-tagging is the most popular and developed approach in imaging biomolecules in living cells. The discovery, cloning, and heterologous expression of the green fluorescent protein (GFP) from the jellyfish *Aequorea victoria* has revolutionized multiple fields in the life sciences, not only in the field of fluorescence imaging [40,42]. Besides GFP, a broad range of FPs that span the entire visible spectrum is now available [43,44]. Genetic engineering further creates photoswitchable FPs that can be both reversibly and irreversibly switched on and off, or converted to different colors [40,41,44,45]. Photoswitchable FPs are useful for super-resolution imaging and monitoring protein diffusion, trafficking, and age [40,41,44,45].

Translational fusion results in the precise labeling of target proteins by FPs. The FPs’ chromophores are generated spontaneously so cofactors other than O_2_ are not required [40]. When applying FP-tagging, it is important to verify the functionality of the fusion protein constructs [46]. It is usually desirable to express the fusion proteins under the control of the native promoters of the target proteins to ensure near native levels of expression.

Small organic fluorescent dyes have enhanced brightness and photostability compared to typical FPs [40,41]. However, they are not genetically encodable and thus lack specificity for any particular protein [40,41]. By using genetic encoded tags, it is now possible to target small organic fluorescent dyes to specific proteins in live cells [41]. Notably, by specifically targeting small organic fluorescent dyes to a nanomachine, Lee *et al.* [47] were able to perform single-molecule super-resolution imaging in live bacterial cells.

#### Photobleaching of Fluorescent Proteins

**Figure 1.**
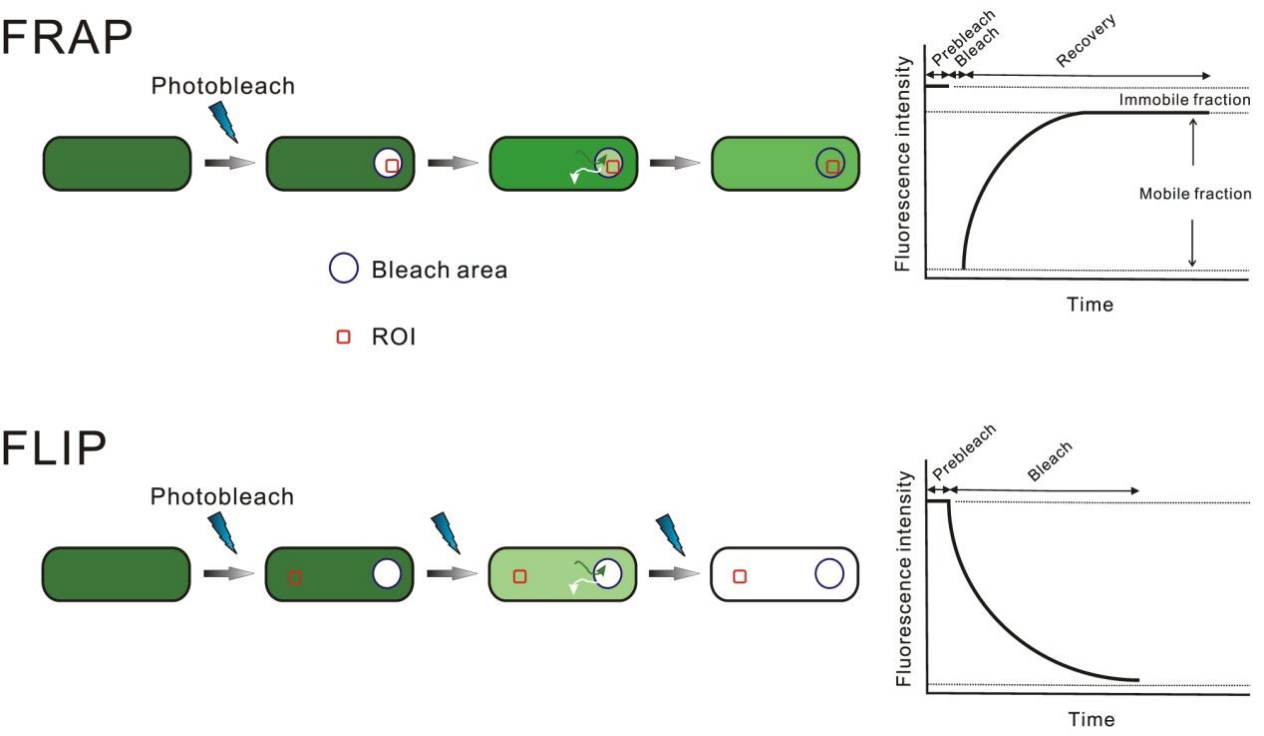
In FRAP, the fluorophores in a region of the cell (blue circle) are selectively photobleached. Fluorescence recovery in that region is assessed quantitatively by monitoring the intensity changes in the region of interest, ROI (red). Kinetics and mobile fraction can be calculated. In FLIP, a region of the cell is photobleached, sometimes repeatedly. The loss of fluorescence from an outside area (red ROI) is monitored. These methods can be used to estimate mobility and kinetic parameters.

Photobleaching techniques are widely used to monitor the kinetics of protein localization/trafficking and of protein–protein interactions in living cells. There are two basic types of photobleaching approaches: fluorescence recovery after photobleaching (FRAP) and fluorescence loss in photobleaching (FLIP) [48] as illustrated schematically in Figure 1. Photobleaching is a photo-induced alteration of a fluorophore that irreversibly extinguishes its fluorescence, most typically due to the generation of free-radicals in the surrounding water solvent which chemically attack the chromophore [48]. In FRAP, a specific region is selectively photobleached with a high-intensity laser and the recovery of fluorescence in this region is monitored over time (Figure 1). The bleach event is preferably as short as possible to avoid fluorescent material diffusing significantly within this initial laser pulse which in effect extends the spatial extent of the bleach zone. The recovery occurs as unbleached fluorophores (e.g., FP-fusion proteins) diffuse back into the bleached region, thus providing a measurement of many kinetic parameters of the tagged protein, including diffusion coefficient, mobile fraction, binding/dissociation rates and the transport rate. Analysis of FRAP kinetics can give important information about the dynamics of protein assemblies like biological nanomachines. The information includes the exchange rates of components, whether the components are bound and released from certain other structures, or whether they exist as monomers or multimers.

Complementary to FRAP, FLIP can be used to examine the continuity of a cellular compartment and the boundaries for a protein’s movement. If fluorophores from an outside area can diffuse into the region being photobleached, loss of fluorescence will occur in the outside area, indicating that the two regions are, in effect, connected. In addition, FLIP can be used to assess the uniformity of a protein’s movement across a particular compartment, and whether there are interactions that impede a protein’s motion. For example, proteins that are stably associated with, and thus constrained by, a cellular structure may photobleach more slowly than those that are freely diffusing. The mobility of various biomolecules within living cells was first measured by using photobleaching techniques. Since then, photobleaching has become the most common means for monitoring protein kinetics *in vivo*. The application of photobleaching techniques to study biological nanomachines *in vivo* has also greatly improved our understanding of their organization and dynamics.

## 3. Super-Resolution and Single-Molecule Fluorescence Microscopy

**Figure 2.**
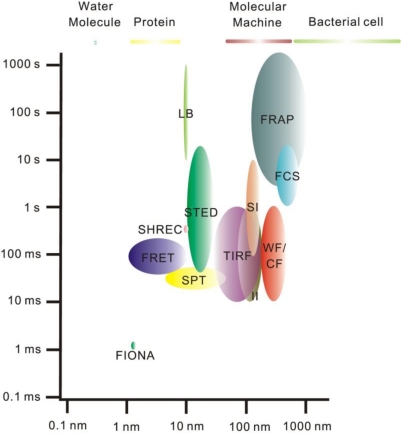
Comparison of the spatial and temporal resolutions of fluorescence microscopy. The length and time scales are logarithmic. Average sizes of some biological features are given (top panel). For FIONA, II, LB, SHREC, SI, SPT, STED, TIRF, and WF/CF, the horizontal dark side of each oval approximates the *x*-*y*-resolution/localization accuracy and the bright side approximates the *z*-resolution/localization accuracy. For example, TIRF and WF/CF have similar *x*-*y*-resolution, but TIRF has a much better *z*-resolution. For FRET the horizontal scale represents distance over which molecular interaction can be detected. For FCS and FRAP the horizontal scale represents the limiting size of the measurement field. The vertical scales refer to the amount of time needed to take one image frame or complete one measurement, the reciprocal of which represents the maximum rate at which dynamic changes in the sample can be detected. FCS, fluorescence correlation spectroscopy; FIONA, fluorescence imaging with one nanometer accuracy; FRAP, fluorescence recovery after photobleaching; FRET, Förster resonance energy transfer; II, interference illumination; LB, localization-based; SHREC, single-molecule high-resolution co-localization; SI, structured-illumination; SPT, single particle tracking; STED, stimulated emission depletion; TIRF, total internal reflection fluorescence; WF/CF, wide-field/confocal. Based on [36,41,58].

**Figure 3.**
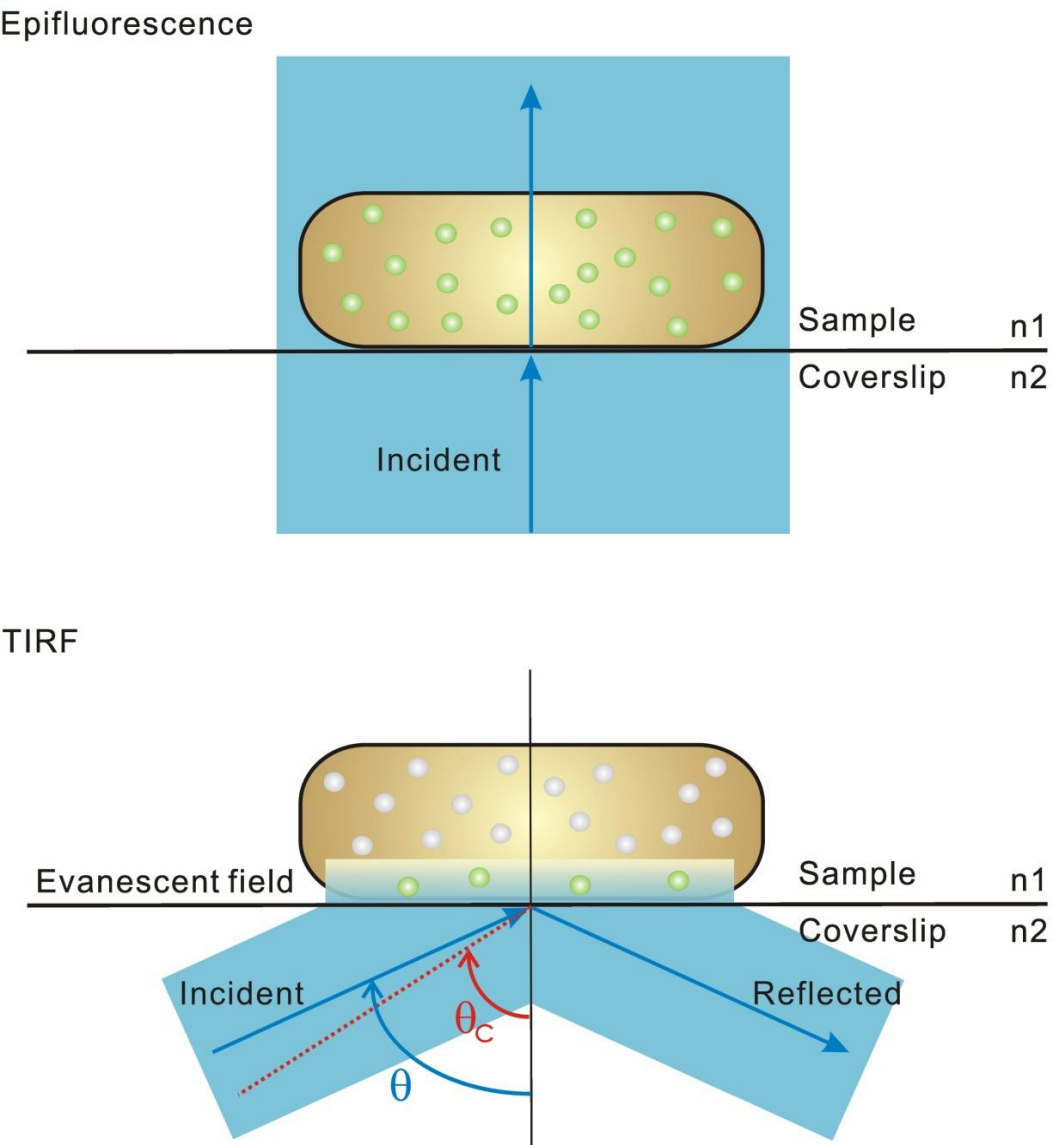
Optical basis of TIRF illumination. In epifluorescence microscopy, the excitation light is transmitted directly through the sample. All of the fluorophores in the entire bacterial cell are excited (green circles). In TIRF microscopy, the excitation light is totally internally reflected from the coverslip/sample interface at the critical angle, θ_c_ (red). When the excitation light travels at a high incident angle θ (blue), which is greater than θ_c_, an evanescent field is generated on the opposite side of the interface. The intensity of the evanescent field decreases exponentially with the distance. Only fluorophores close to the surface are significantly excited. To achieve TIRF, the refractive index of the sample (n_1_, typically 1.33 for water-based pH buffers, with the cell itself having a slightly higher index of *ca.* 1.35) must be less than that of the coverslip (n_2_, typically 1.52 for glass).

The diffraction-limited optical resolution for so-called far-field optical microscopy is *ca.* 200–300 nm, which was once regarded as an unbreakable barrier. A variety of super-resolution “nanoscopy” techniques, which effectively break the optical resolution limit now exist and permit observation of individual proteins and complexes that are on the length scale of *ca.* 1–50 nm [33,49] (Figure 2). These super-resolution methods image protein localization and dynamics at near-molecular length scales [33,34,36,41,45,49–54]. These methods can generally be categorized as near-field or far-field approaches; for the former either the detector is placed a distance less than the optical resolution limit away from the fluorescence emitter, as in scanning near-field optical microscopy (SNOM) for example, or the excitation field itself extends spatially less than the optical resolution limit [33]. Total internal reflection fluorescence (TIRF) microscopy is the most prominent near-field excitation approach, which utilizes the effect of total internal reflection, generally now involving a laser beam directed at a highly inclined angle to typically a glass–water interface for the illumination of fluorophores (Figure 3) [33,36,55]. TIRF generates an exponentially decaying evanescent field at the interface of the two media of different refractive indices (such as the glass of a coverslip and the water-based media of a pH buffer surrounding a cell), which can selectively excite fluorophores in cells near a thin region extending to *ca.* 100–200 nm beyond the surface of the coverslip (*i.e.*, the excitation volume is delimited parallel to the vertical axis) [33,36,55]. Consequently, there is very little fluorescence signal contributed from fluorophores, or contaminants, beyond this thin optical slice, so the signal-to-noise ratio can be exceptionally high, permitting single-molecule detection. It is a technique of choice for studying functional nanomachines that are expressed in cell membranes and therefore within reach of the thin excitation volume. Although the lateral resolution of TIRF is diffraction-limited at 200–300 nm, the axial resolution is set by the extent of the evanescent excitation field and so may be less than the optical resolution limit. Thus TIRF mircoscopy is rightly regarded as a super-resolution method.

**Figure 4.**
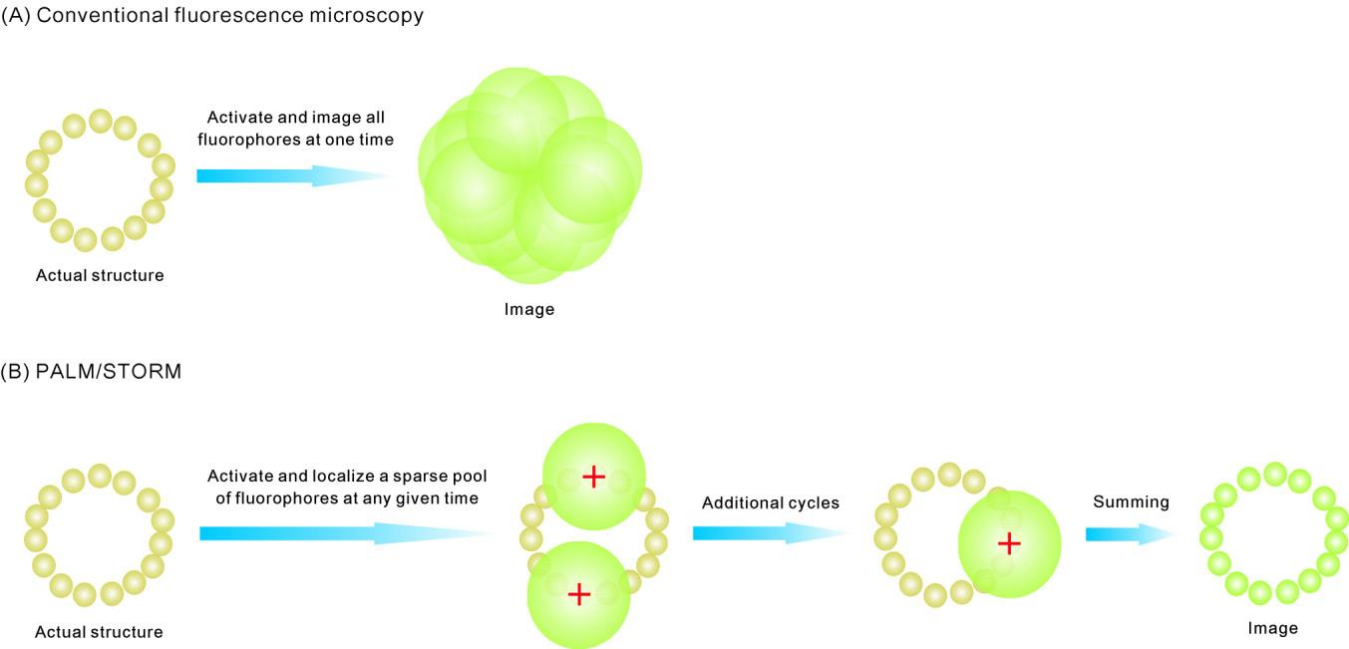
Principles of PALM/STORM. (**A**) In conventional fluorescence microscopy, all fluorophores are excited at once, thus the diffraction-limited areas from closely positioned fluorophores overlap; (**B**) PALM/STORM excites only a small subset of fluorophores at any given time, so the diffraction-limited areas of each fluorophore no longer overlap. The precise location of each fluorophore can be determined by finding its intensity centroid from the detected fluorescence image. By repeating the cycle, the many measured locations of distinct fluorophores in the entire cell are superimposed to generate a final super-resolution image.

Various pure far-field approaches are available for super-resolution and single-molecule fluorescence imaging, namely localization-based, stimulated emission depletion, structured-illumination, and non-linear optical methods [33,36,49,50]. Among them, single-molecule localization microscopy (PALM/STORM) detects fluorescence emitted from a single fluorophore (or a small number of fluorophores) and subsequently determines the molecule’s position (Figure 4) [33,36,45,49,50,52,56]. Taking advantage of the fact that the point spread function (PSF) of a microscope can be precisely determined, the intensity centroid from a fluorescence emitter (*i.e*., the exact position of the fluorophore) can be localized to nanometer-scale accuracy [33,34,36,45,49,50,52,56,57] provided a sufficient number of emitted photons can be sampled. In each imaging cycle a small, optically resolvable fraction of fluorophores are imaged.

With repetitive imaging cycles, the positions of a high proportion of fluorophores in the sample may ultimately be determined, allowing the reconstruction of an extended super-resolution image, provided the *ca.* 200–300 nm diameter diffraction-limited PSF images from neighboring single fluorophores do not overlap in a given cycle (*i.e*., the effective active fluorophore concentration in the cell is kept sufficiently low at any given time [31,59]). If an FP is used as the fluorophore, this can be achieved in many ways [59]. Generally, the expression of the target protein-FP fusion is controllable, and the emitting FP population can be reduced via photobleaching before measurements are made [59]. Moreover, photoswitchable/photoactivatable FPs can be used [34,45]. The emission of these FPs can be switched on and off under the control of light. The activation light (often in the ultraviolet) may illuminate the entire sample at a low intensity, so only one or a few FPs are activated in a stochastic fashion into an activated conformation that can then be excited subsequently into fluorescence by a second light source of higher wavelength. The localization accuracy is dependent on how bright (the number of photons detected) the FP is over the background signal (cell autofluorescence and camera detector readout and pixilation noise, as well as the dim fluorescence of non-active FPs emitting in the activation channel which is often the largest component) [34,45,52,56].

The performance of PALM/STORM depends critically on the labeling density and the biological structure under investigation [33]. It may perform better for imaging smaller or filamentous objects than dense and bulky structures [33]. The main weakness is the need to collect, process, and integrate many raw images which may take a long time, and so this is not particularly useful for monitoring very fast, dynamic processes [36]. However, the relatively simple optical setup has led to its rapid adoption. New improvements also have largely reduced the time required for generating an image. Conventional single-molecule tracking experiments require low densities of target molecules. PALM/STORM using photoactivatable/photoswitchable fluorophores allow a high density of target molecules to be labeled and tracked. Although it may still take a substantial time to collect the large number of localizations required to construct a high-resolution extended structure, the motion and dynamics of the molecules inside the structure can be obtained with *ca.* millisecond temporal resolution from the single-molecule tracking. Indeed, several studies have used the PALM/STORM approach to visualize protein movements and dynamic events in living cells [49,50]. This approach also has a great potential to probe the organization and stoichiometry of molecular complexes.

Among the different single-molecule localization approaches, ***f***luorescence ***i***maging with ***o***ne ***n***anometer ***a***ccuracy (FIONA) [52,57,60] is of immense potential for single-molecule studies. This technique can locate the position of a fluorophore with accuracy in the range of a few nanometers. By using FIONA, Kural *et al*. [61] located GFP–tagged peroxisomes in cultured *Drosophila* S2 cells to within 1.5 nm with a time resolution of just 1.1 ms. They found that the peroxisomes are moved by dynein and kinesin in 8.3-nm steps. By applying FIONA to samples labeled with two spectrally different fluorophores, one can reach ***s***ingle-molecule ***h***igh-***re***solution ***c***o-localization (SHREC) which can measure relative separations of the different colored fluorophores larger than *ca.* 10 nm [52,62–64]. Currently, SHREC is effective when the number of fluorophores is limited, and has only been applied with great success to study myosin V, and for studying properties of the kinetochore [65,66]. There is also related techniques called SHRImP [67] and NALMS [68], which were invented independently but are essentially the same technique; they use photobleaching to localize two closely placed fluorophores to nanometer accuracy, applied for example to study the hand-over-hand action of myosin VI [69], but now capable of sub-nanometer accuracy [70].

Single-particle tracking (SPT) has been applied to visualize the movements of single (or small numbers of) molecules in live cells by optical microscopy. The development of single-molecule tracking photoactivated localization microscopy (sptPALM) can contribute to a quantitative understanding of the dynamics of individual molecules, and provide new insights into the mechanisms of many biological processes, including protein heterogeneity in the plasma membrane, the dynamics of cytoskeletal systems, and clustering of receptor complexes [71]. SPT and sptPALM have both been applied to several bacterial systems. For example, the localization mechanism of the *Caulobacter crescentus* histidine kinase PleC and the pole-organizing protein PopZ was shown to be consistent with a diffusion-to-capture model by SPT [59,72,73].

**Figure 5.**
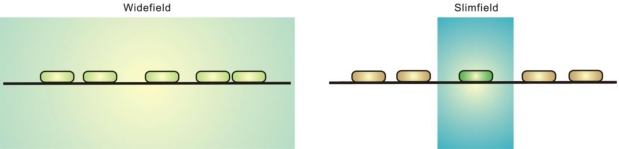
Slimfield illumination. Conventional wide-field fluorescence microscopy illuminates a wider field of the sample by lower excitation intensity, while slimfield microscopy concentrates the excitation light into a smaller area with greater excitation intensity.

Temporal resolution is another factor to be considered for super-resolution fluorescence microscopy methods. To overcome the challenge of imaging fast events in living cells, several methods have been developed, including slimfield microscopy, brighter fluorophores, and new millisecond or even sub-millisecond time-resolution cameras [22,49]. Slimfield microscopy (Figure 5) utilizes a different illumination mode suitable for rapid (millisecond) temporal resolution. By concentrating the excitation light into a small area (∼30 μm^2^), slimfield microscopy illuminates the sample with excitation intensities ∼100 times greater than those of conventional wide-field fluorescence microscopy [74]. The much greater excitation intensity overcomes camera noise when lowering the frame integration time to millisecond levels, permitting single-molecule detection at high speed [74]. It is also possible to perform simultaneous dual-color slimfield imaging for co-localization and FRET studies [74].

Stroboscopic illumination can provide another means of enhancing temporal resolution of live-cell single-molecule imaging to sub-millisecond levels [31]. In this method, a short laser pulse can overcome the limitation of a slow mechanical shutter or slow frame rate due to large pixel arrays of the CCD. The shutter and the CCD can be left open for longer times while an intense excitation laser pulse for a short duration is applied, during which the FP-fusion construct under study does not diffuse significantly. Thus the temporal resolution in stroboscopic illumination is determined by the laser pulse width. By varying the pulse width and dwell time dynamic properties such as residence times of weak binding and diffusion constants can be estimated. The main drawback of this method is that single FP molecules are more photolabile under the high pulse intensity. This approach has been used to image single cytoplasmic FP and fast diffusion of single transcription factors in live bacterial cells [31].

## 4. Experimental Studies

### 4.1. Flagellar Motors

The bacterial flagellum is a complex nanomachine powered by H^+^ or Na^+^ ion flux [7,75]. It is a large membrane-spanning structure generated from choreographed expression and assembly of ∼50 genes. Each flagellum consists of a filament, a hook, and a basal body. The flagellar motor in the basal body has a stator unit that pushes a rotor at several hundred Hz for the H^+^-driven motor, and up to a few kHz for the Na^+^-driven motor. In many bacterial species, the motor can switch its direction, with the switching rates controlled by chemotatic signaling [7,75].

**Figure 6.**
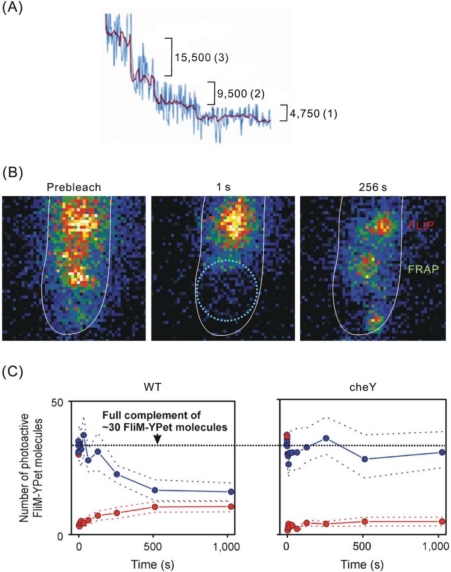
Stoichiometries and dynamics of a functioning flagellar nanomachine in living bacterial cells. (**A**) Stepwise photobleaching of GFP–MotB molecules in a motor after prebleaching of the cell to reduce background. Shown are the raw motor intensity (blue), Chung-Kennedy filtered motor intensity trace (red), and detected steps (in parentheses) and step sizes; (**B**) Successive TIRF images of a cell region before and after photobleaching. The boundary of the laser focus is indicated (dotted circle, middle panel). FLIP (red) and FRAP (light green) of GFP–MotB molecules of two motors are shown. The cell is outlined (white) [76]; (**C**) Turnover of FliM molecules in the switch complex is dependent on the response regulator CheY. Mean FRAP (red) and FLIP (blue) traces (dotted lines: SEM error bounds) based on 7–11 spots are shown for FliM-YPet in WT and Δ*cheY* cells [77].

In a pioneering study, Leake *et al*. [76] used TIRF mircoscopy and photobleaching to obtain the stoichiometry of the flagellar motor nanomachine in live bacterial cells. In addition, FRAP and FLIP were utilized to complement super-resolution/single-molecule imaging and SPT for studying the dynamic movement of the stator component MotB in the protein complex. By attaching living *Escherichia coli* cells via one of their flagellar filaments to a coverslip, rotation of cells, an indication of the functionality of the GFP–MotB fusion protein, was observed simultaneously with fluorescence emissions. Taking advantage of a phenomenon of stepwise/discrete photobleaching (fluorophores like GFP will photobleach stochastically in a step-like manner), the stoichiometry of the stator component MotB (Figure 6A) in functioning flagellar motors was then estimated in these live *E. coli* cells. Each flagellar motor contains ∼22 MotB molecules. Since it was known that two MotB molecules form a single stator unit, there are thus *ca.* 11 stator units per motor. FRAP and FLIP studies showed that MotB diffuses in the membrane when it is not incorporated into a motor. Motor-integrated MotB molecules are turned over and exchanged with a membrane pool (∼200 molecules) about once every 30 seconds (Figure 6B). Importantly, this was the first direct measurement of the stoichiometry, dynamics and turnover of protein subunits within a functioning molecular machine.

A subsequent study using similar approaches has shown that an *E. coli* flagellar motor contains ∼30 copies of the rotor switch component FliM (Figure 6C) [77]. These FliM molecules exist in two discrete populations, one tightly associated with the motor (*ca.* 1/3 of all motors) and the other undergoing stochastic turnover (*ca.* 2/3 of all motors; half-life: ∼40 s). These may reflect two previously described populations of FliM, exchangeable FliM in a peripheral location and FliM located in the core of the complex [78,79]. Importantly, it was found that the turnover of FliM molecules depended on the presence of activated response regulator CheY (Figure 6C), which binds FliM to cause a switch in rotational direction of the motor [77]. The work of Delalez *et al*. [77] provides direct evidence for chemotatic signal-dependent dynamic exchange of a switch complex component in functioning flagellar motors. Thus the exchange of FliM subunits could be either a cause or effect of motor reversal [77,80]. Moreover, there are ∼24 FliM spots per cell, 2–8 times more than the typical number of complete flagella. Of all fluorescent FliM spots observed, 40% have ∼30 FliM molecules per spot, 60% have ∼18 FliM molecules. The 18-molecule spots may represent preassembly C rings that have not fully integrated into functional motors. It is estimated that there is a total of 630 ±290 FliM molecules per cell, with a comparable number of FliM molecules diffusing freely. The estimated total number of FliM molecules is in very good agreement with that measured from earlier biochemical studies, indicating the power of single-molecule imaging in determining the *in vivo* stoichiometries of biological nanomachines [77]. In addition, this study suggests that protein turnover and exchange may play active roles in the function of biological nanomachines, and not only a passive role in the maintenance of macromolecular complexes.

### 4.2. Membrane Transporters and Energetic Complexes

About 25% of a cell’s proteome must translocate across membrane [81]. In bacteria, the Sec pathway transports proteins as unstructured linear peptide chains across the cytoplasmic membrane, whereas the protonmotive force (PMF)-driven twin-arginine translocation (Tat) system translocates folded proteins [15,81,82]. Since folded proteins are larger and more variable in size, the translocation is particularly challenging for the Tat transport nanomachine. Indeed, the exact mechanism by which the Tat system transports proteins is under debate. At least three models have been proposed: (1) the polymerization model, in which substrate interaction with TatBC triggers TatA polymerization; (2) the bespoke channel model, in which the dynamic variation of TatA oligomeric state could maintain a seal around substrates of different sizes during transport; (3) the bilayer perturbation model, in which TatA polymerization may alter local membrane bilayer structure to allow substrate movement [15,83]. In *E. coli*, three integral membrane proteins TatA, TatB, and TatC are essential Tat components [81]. TatB and TatC are involved in signal peptide recognition and the targeting of protein substrates to TatA. Previous structural and biochemical information suggests that TatA forms a ring-shaped translocation pore of the Tat system [81].

To better understand the mechanism of Tat-mediated protein translocation, it is critical to determine the oligomeric state and organization of TatA *in vivo* directly. To this end, Leake *et al*. [83] applied stepwise photobleaching to measure the stoichiometry of TatA–YFP in a native membrane environment. Custom-written software automatically identified and tracked TatA spots through stacks of consecutive images, which determined the intensity of each TatA complex together with its position to a precision between ∼2 and ∼20 nm. They found that TatA forms ∼15 mobile complexes with ∼25 TatA sub-units per complex. The mobility of TatA complex decreases with increasing complex size. Analysis of the stoichiometry distribution suggests the TatA complexes are assembled from tetramer units. There are ∼460 TatA molecules associated with complexes in a cell, with ∼100 molecules of a disperse membrane pool. Notably, mathematical modeling of the diffusion behavior of the complexes indicates that TatA protomers organize into a ring and not a filled bundle/disc. Loss of PMF has no effect on the stoichiometry of TatA complex. However, TatA does not form complexes in Δ*tatBC* cells, suggesting that TatBC controls the oligomeric state of TatA. The mechanistic models of Tat transport make specific predictions about the oligomeric state of TatA and whether and how this changes during the transport cycle. Although previous biochemical studies have shown that TatA exhibits heterogeneous oligomeric states, it is uncertain whether this heterogeneity is an artifact of the detergent extraction [81]. Leake *et al*. [83] demonstrated that this variability in oligomer size is an inherent property of TatA *in vivo*, with a polymerization TatA complex model being most likely.

Bacterial nanomachines work in a whole cellular context. Therefore, the subcellular distributions of these nanomachines are important for a comprehensive understanding of how they function. Nearly all organisms are able to synthesize ATP by OXPHOS (oxidative phosphorylation), a biochemically well-understood process carried out by many membrane-bound enzymes [84]. However, little was known until recently about the relationship between its functions and cellular spatial localization.

By tagging OXPHOS ATP synthase and succinate dehydrogenase with different fluorescent proteins, Johnson *et al*. [85] showed that these complexes distribute heterogeneously in mobile patches in living *Bacillus subtilis* cells. However, the dynamic localization of the complexes was investigated at relatively low temporal resolution and the stoichiometry was not quantified. Lenn *et al*. [13,86] further studied the organization and dynamics of functional GFP-tagged cytochrome *bd*-I oxidase complex in living *E. coli* cells. TIRF microscopy, SPT and stepwise photobleaching showed that ∼76 cytochrome *bd*-I oxidases cluster into mobile spots in the cytoplasmic membranes. The positions of these clusters were obtained to within a few nanometers precision. Like TatA, cytochrome *bd*-I clusters are assembled from tetramer units. There are ∼230 clusters per cell, and ∼88% of all cytochrome *bd*-I oxidases are directly associated with the clusters. Interestingly, the cluster widths (with a mean of ∼100 nm) of cytochrome *bd*-I clusters do not increase with cluster intensities, indicating that the diameters of the clusters are not determined solely by the number of constituting cytochrome *bd*-I complexes. The authors suggested that respiration occurs in mobile membrane clusters which they called “respirazones”. These specialized compartments are dedicated to respiratory function. Within the putative respirazones, OXPHOS complexes and electron carriers might be highly concentrated, thus enhancing energetic efficiency. In OXPHOS, the oxidation of electron donors is coupled with the generation of PMF. If ATPases and proton symporters/antiporters are also associated with respirazones, the efficiency of PMF-dependent processes could also be enhanced. Intriguingly, the adventurous gliding motility of the Gram-negative bacterium *Myxococcus xanthus* is mediated by the rotation of a helical cytoskeleton powered by PMF [87]. The motility cytoskeleton interacts with MotAB homologs. A mechanochemical model was proposed: PMF-driven motors (similar to flagellar stator complexes) run along a helical track and drive the rotation of the track, thus pushing cells forward. Further co-localization studies of OXPHOS components with PMF-driven motors could provide insights of whether there are localized power supplies for different PMF-dependent processes in the cytoplasmic membrane [13,86].

PspA is a peripheral membrane protein which maintains PMF under stress conditions [88]. *In vitro* studies have shown that PspA exists in oligomeric states [88,89]. However, the stoichiometry of the PspA complexes *in vivo* is undetermined. Lenn *et al*. [90] utilized wide-field fluorescence microscopy and photobleaching to measure the stoichiometry of PspA complexes in living *E. coli* cells. Their results indicate that PspA may mainly exist as hexamers in the cytoplasmic membrane. They show that quantifications with single-molecule sensitivity can be achieved under normal epifluorescence illumination. Engl *et al*. [91] have shown that PspA is organized into two distinct functional classes, one localizes at the cell pole and the other at the lateral cell membrane. The highly mobile lateral PspA complexes are absent in cells lacking the MreB cytoskeleton, and cells fail to maintain PMF under stress conditions. Interestingly, the *M. xanthus* PMF-driven motility system is also dependent on the MreB cytoskeleton [87].

### 4.3. The Replisome

The bacterial replisome is a multiprotein nanomachine that replicates DNA at a rate ∼1000 nucleotides per second and makes less than one mistake per 10^9^ nucleotide incorporations [92]. The robust and efficient coordination of its components accounts for the high efficiency and fidelity. Although the replisome has been extensively studied *in vitro* [92,93], its *in vivo* composition and supramolecular architecture has been a mystery until recently. For a cytoplasmic protein complex, the replisome itself has a relatively slow apparent diffusion, with a mean-square displacement (MSD) of ∼10^3^ nm^2^/s [94], because it is bound to the nucleoid DNA. However, with non-bound cytoplasmic components diffusion faster by a factor of *ca.* 1000, at a comparable rate to the replication of the DNA bases themselves. Conventional video-rate (tens of milliseconds per image frame) fluorescence microscopy that has been used to study membrane-integrated protein complexes is not sufficient for studying these fast dynamics of the replisome without running the risk of generating blurry images. By using slimfield fluorescence microscopy and stepwise photobleaching, Reyes-Lamothe *et al*. [95] determined the stoichiometry of the replisome. The authors tagged the *ca.* 10 replisome components with the fluorescent protein YPet. The fusion proteins were under control of the native promoters.

**Figure 7.**
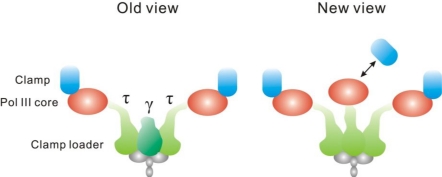
Models for replisome composition/architecture. The old view is based on previous *in vitro* experiments, and the new view based on the work of Reyes-Lamothe *et al*. [95]. Components that are identical in both models are shown in gray. In the new model, there are three Pol IIIs, three β clamps (one of them is dynamically localized, either within or outside the replisome core), and three τ subunits.

They observed ∼75% of cells contain two spatially separated replisomes (*i.e*., two fluorescent spots), each associated with independent replication forks. The remaining ∼25% of cells have sister replisomes separated by a distance smaller than the diffraction limit of the imaging system and are seen as a single fluorescent spot. Correspondingly, most replisome components have bimodal 1:2 distributions of stoichiometries, with single fluorescent spots displaying doubled stoichiometries. Earlier *in vitro* experiments established a textbook view of DNA polymerase III (DNA pol III): each replisome containing two pol IIIs (Figure 7), one for a replication fork on the leading strand and one for the lagging strand [14]. Surprisingly, Reyes-Lamothe *et al*. [95] found that each replisome contains three pol IIIs (Figure 7). The clamp loader that links pol IIIs also has three copies per replisome. However, the spatial intensity distribution of a clamp component suggested that only ∼27% of replisomes have all three clamps associated with the Pol III core. In other words, in ∼73% of replisomes one clamp localizes outside this core. These results indicated that in a few cells all three pol IIIs may be associated with active replication, while in most cells the third pol III may be waiting to be loaded on to the lagging strand. The functional insight of this architecture might be that the third Pol III facilitates the replication of the lagging strand [95,96]. In combination with degron-targeted proteolysis and gene deletion of specific proteins, Reyes-Lamothe *et al*. [95] also obtained an unanticipated insight into the architecture of the replisome by *in vivo* single-molecule imaging. They showed that the single clamp loader in each replisome contains three τ proteins but no γ, instead of the previously believed two τ subunits with a single γ subunit (Figure 7) [14]. By tracing single YFP-labeled primases, events of transient binding and unbinding of individual primases at the replisome that possibly correspond to the formation of Okazaki fragments was also observed in living *E. coli* cells [31].

## 5. Cytoskeletons

### 5.1. MreB

MreB, the most widely conserved prokaryotic actin homolog, is found in almost all non-spherical bacteria [6,97]. MreB homologs have been shown to form filaments with biochemical and structural properties strikingly similar to those of actin, and been implicated in diverse cellular spatial regulations, including chromosome replication, segregation, and decatenation, cell growth and division, morphogenesis, polarity, protein localization, organelle positioning, and differentiation [6,97]. MreB homologs assemble into helical filaments beneath the cell membrane or ring-like structures at putative division sites [6,97].

Combinations of single-molecule imaging and SPT/sptPALM have yielded plenty of new information about the MreB cytoskeleton. When 3–4 MreB-YFP fusion proteins were expressed in each *C. crescentus* cell under the control of an inducible promoter in a background of wild-type MreB, polymerized and unpolymerized MreB monomers were distinguished [59,98]. Unpolymerized MreB molecules show Brownian diffusion that is slower than expected for a cytoplasmic protein but is consistent with the motion of a membrane-associated protein, whereas polymerized MreB molecules display slow, directed motion (average speed: 6.0 ± 0.2 nm/s). Importantly, this directional movement of MreB in the growing polymer provides an indication that, like actin, MreB monomers treadmill through MreB filaments by preferential polymerization at one filament end and depolymerization at the other filament end [59,98]. It was suggested that MreB helices may serve as tracks for the movements of other cellular components. However, single MreB filaments are much shorter (392 ± 23 nm) than the cell length and the direction of their polarized assembly seems to be independent of the overall cellular polarity. Thus, the long helical MreB structures that have been visualized represent bundles of short filaments, and these helices lack a uniform global polarity [59,98]. PALM was further used to explore the super-resolution structure of the MreB cytoskeleton to a precision of *ca.* 40 nm. After the photobleaching of all emissive YFP-MreB molecules, a sparse subset was reactivated in each diffraction-limited region [59,99]. Two distinct MreB superstructures were identified in *C. crescentus*: a quasi-helical arrangement in a stalked cell and a mid-cell ring in the pre-divisional cell [34,59,99]. Uniquely, by using the natural treadmilling motion of MreB, the number of localizations (thus the effective resolution) was increased without further MreB induction [34,99]. Since each localization event (*i.e*., each determination of a position) along the MreB cytoskeleton comes from a single 100 ms frame in these studies, multiple position determinations may come from a single MreB-YFP molecule as it treadmills through an MreB protofilament. This approach allowed researchers to use a smaller real concentration of FP-fusions to obtain a large number of spatial localizations. Live-cell dynamics can thus be utilized to obtain higher resolution information of subcellular architecture [34,99].

### 5.2. FtsZ

FtsZ, a tubulin homolog, is the major component of the bacterial cytokinesis nanomachine [6,8]. It is almost universally present in bacteria as well as in the chloroplasts and mitochondria of some eukaryotes. FtsZ forms the Z-ring under the membrane at the mid-cell, and this cytoskeletal structure serves as a scaffold to recruit and position a cascade of proteins that have diverse functions in cell division and cell wall synthesis. The constriction of the Z-ring initiates cytokinesis. However, whether the Z-ring is a passive scaffold or an active force generator directing inward growth of the cell wall has long been debated [6,8]. Super-resolution and single-molecule imaging is essential to explore this and other important aspects of FtsZ functions *in vivo*.

Niu and Yu [100] applied sptPALM to investigate the dynamics of FtsZ in live *E. coli* cells. They found two subpopulations of FtsZ molecules with distinct diffusion dynamics. The FtsZ molecules forming the Z-ring are mainly stationary, and the rest of the unpolymerized FtsZ molecules undergo Brownian motion spanning the whole cell. Intriguingly, the diffusion of FtsZ is spatially restricted to helical-shaped regions. Fu *et al*. [101] further used PALM to characterize the *in vivo* structure of the Z-ring in *E. coli*. They found that in addition to the ring-like conformation, the Z-ring also adopts a novel compressed helical conformation with variable helical length and pitch. The conformation of the Z-ring is dependent on FtsZ expression level. The thickness of the Z-ring is ∼110 nm. These results suggest that the Z-ring is composed of a loose bundle of FtsZ protofilaments that randomly overlap with each other in both longitudinal and radial directions of the cell. Similar observations were made by super-resolution imaging based on STED microscopy [102]. The above information provides important insights for the investigations of structure-function relationships and spatial regulation of the FtsZ cytoskeleton.

### 5.3. ParA

The ParABS system is long regarded as a mitotic-like apparatus for active and faithful partitioning of plasmid and chromosomes in bacteria [9]. This mitotic-like force generation device provides a means for the identification of DNA cargos and a way to move them. The *parABS* locus contains a *cis*-acting DNA region *parS* (centromere) and encodes two *trans*-acting proteins: the ATPase cytoskeletal protein ParA (motor) and a centromere-binding protein ParB that interacts with the ATPase (adaptor) [9]. Emerging evidence indicates that ParABS systems encoded by chromosomes and plasmids segregate DNA by similar mechanisms [9]. Shared features of chromosomal and plasmid ParAs are: (1) dynamic movement over the nucleoid; (2) ATP-dependent non-specific binding to DNA; (3) interaction with the ParB–*parS* complex [9,103,104]. Despite these shared features, the actual molecular mechanisms linking ParA dynamics with DNA partitioning is still unknown [9,103]. Basically, two types of models are proposed: a filament-pulling model and a diffusion-ratchet model [103]. Both models employ a “time-delay ratchet” which may be originated from the slow multi-step conformational transition of ParA upon ATP binding [103,104]. The primary difference between these models is whether ParA polymerizes into filaments, and whether depolymerization of filaments can provide the pulling force for DNA segregation [103]. It is thus critical to clarify whether ParA really polymerizes into filaments *in vivo* to understand the mechanism of ParA-mediated DNA partitioning.

By utilizing super-resolution fluorescence imaging, Ptacin *et al*. [104] provide direct evidence that ParA forms slightly curved cytoskeletal filaments in *C. crescentus* cells. The “comet tail-like” polarized ParA gradients observed in different chromosomal and plasmid ParABS systems under diffraction-limited fluorescence microscopy probably correspond to narrow linear ParA structures that have widths of 40.1 ± 9.5 nm [104]. In the burnt-bridge Brownian ratchet filament-pulling model, a pulling force is generated by depolymerization of ParA filament that is stimulated by the ParB–*parS* complex [9,103,104]. The moving ParB–*parS* complex “ratchets” along the end of a retracting ParA filamentous structure and leaves behind it a ParA-free nucleoid zone. The ParB–*parS* ratchet utilizes the slow multi-step conformational transition of ParA to ensure the directionality of DNA movement. A retracting ParA cytoskeleton can pull the chromosome with a speed of ∼120 nm/min [105]. Ptacin *et al*. [104] combined super-resolution and conventional fluorescence imaging, genetic analysis, and biochemical approaches to support this model. Their results demonstrate that the operating principles of the filament-pulling mechanism are similar to eukaryotic mitotic machinery: a multivalent protein complex at the centromere stimulates the dynamic disassembly of cytoskeletal filaments to move DNA into daughter compartments. Significantly, there is growing evidence that similar ParA-dependent systems are used in several other partitioning processes other than DNA segregation in bacteria [106–108]. It is possible that ParA-dependent systems represent a family of universal partitioning machinery.

## 6. Conclusions

In addition to the studies of nanomachines, single-molecule and super-resolution fluorescence imaging have been applied to explore many other cellular events in living bacterial cells. From these studies, a hitherto undiscovered picture of the cell has gradually emerged [22,31,39,59,109]. Soon *in vivo* single-molecule and super-resolution fluorescence imaging will become a standard tool in investigating molecular processes underlying diverse biological phenomena. The abilities of visualizing and quantifying individual molecules at work with spatial distributions and temporal resolutions in living cells offer a “bottom-up” approach for systems-level understandings [22,30,110]. Indeed, fluorescence microscopy is a promising method that could be a vital tool for *in vivo* high-throughput studies [30,110]. Although systems biology has explosively uncovered many new cellular networks, a comprehensive understanding of a biological nanomachine with only five components (ParA, ParB, *parS*, ATP, and cargo DNA) is still beyond current technical experimental feasibility [103]. We believe that single-molecule and super-resolution fluorescence imaging is the key to reveal the hidden behaviors of biological nanomachines and other cellular systems. Developments in multicolor single-molecule fluorescence imaging and optogenetics [111,112], the method of controlling cellular functions with light, will enormously increase our ability to observe and manipulate nanomachines in their physiological context.

Let there be light!
